# A Phase 2b randomized clinical trial of the oral p38α inhibitor neflamapimod in dementia with Lewy bodies (DLB)

**DOI:** 10.21203/rs.3.rs-10623740/v1

**Published:** 2026-08-14

**Authors:** John-Paul Taylor, John J. Alam, Stephen N. Gomperts, Lawrence S. Honig, Niels D. Prins, Isamil Koubiyr, Hui-May Chu, Amanda Gardner, Kelly Blackburn, Menno M. Schoonheim, Charlotte E. Teunissen, James E. Galvin

**Affiliations:** Newcastle University; CervoMed, Inc; Massachusetts General Hospital, Harvard Medical School; Columbia University; Brain Research Center; Amsterdam Neuroscience, Amsterdam UMC, Vrije Universiteit Amsterdam; Anoixis Corporation; CervoMed, Inc; CervoMed, Inc; Amsterdam Neuroscience, Amsterdam UMC, Vrije Universiteit Amsterdam; Amsterdam Neuroscience, Amsterdam UMC, Vrije Universiteit Amsterdam; University of Miami Miller School of Medicine

**Keywords:** Dementia with Lewy bodies, phase 2 clinical trial, neflamapimod, basal forebrain atrophy, cholinergic dysfunction and degeneration, Alzheimer's disease co-pathology

## Abstract

**Background:**

There is high unmet need in dementia with Lewy bodies (DLB). Neflamapimod, showed clinical activity against cognitive and motor endpoints at a dose of 40mg three-times-daily (TID) in a prior phase 2a study in DLB, most prominently in those without Alzheimer disease (AD) co-pathology, assessed by plasma ptau181.

**Methods:**

16-week randomized, placebo-controlled clinical trial, with a 32-week open label extension, conducted at 43 centers in the US, UK and Netherlands. 159 participants with probable DLB by consensus criteria, screened to enrich for patients without AD co-pathology (plasma ptau181≥27.2 pg/mL excluded), randomized between August 2023 and June 2024 to neflamapimod 40mg (80 participants) or matching placebo (79 participants) capsules, TID. Mean age 71.4 (6.1), 85.6% male. Primary outcome measure: change in Clinical Dementia Rating Sum-of-Boxes (CDR-SB). Secondary outcome measures: Timed up and Go test, ADCS Clinical Global Impression of Change, and a study-specific cognitive test battery. Biomarker measures: plasma levels of glial fibrillary acidic protein and neurofilament light chain, and basal forebrain atrophy by structural MRI (sub-study in 18 participants).

**Results:**

There was no significant difference between neflamapimod and placebo on the primary outcome measure, change in CDR-SB. There were also no differences seen on secondary clinical outcome measures and the two plasma biomarkers, though in the sub-study, improvement relative to placebo was seen with neflamapimod on change in right basal forebrain volume from baseline to week 16. In exploratory analyses, the treatment group difference in change in CDR-SB favored neflamapimod compared to placebo among participants with low (<21 pg/mL) screening plasma p-tau181. Pharmacokinetic data showed that mean trough neflamapimod plasma drug-concentrations (C_trough_) were lower than expected, and treatment effect favoring neflamapimod was seen in participants with C_trough_ above the study median. Discontinuation due to adverse events was 2.5% (2 of 79) with neflamapimod and 1.3% (1 of 80) with placebo

**Discussion:**

The phase 2a clinical study findings were not replicated, potentially due to the inclusion of a higher proportion of patients with AD co-pathology than targeted and the use of neflamapimod drug product that did not achieve expected plasma drug concentrations.

Registered at clinicaltrials.gov on 17 May 2023 (NCT0586966)

## BACKGROUND

There is a high unmet medical need in dementia with Lewy bodies [^1,2,3^], with the standard-of-care therapy, cholinesterase inhibitors, providing only mild symptomatic improvement in cognitive function, fluctuations and visual hallucinations, and no benefits (with some possible detriments) on motor function [^4,5^]. From a pathogenesis standpoint, DLB is an a-synucleinopathy that in its early stages particularly affects the basal forebrain cholinergic system [^6,7,8^], the primary source for cortical neurotransmitter acetylcholine. Many patients with DLB, particularly in the elderly, also often develop biomarker evidence of amyloid and/or tau pathology, which may occur prior to or coincident with, development of hippocampal atrophy [^9,10^]. Blood-based biomarkers, including plasma tau phosphorylated at position 181 (p-tau181), has been shown to correlate with this progression [^11^], with increased levels correlated with cognitive decline, AD co-pathology, and more extensive neurodegeneration [^12,13^].

Neflamapimod is an oral p38α kinase inhibitor that targets molecular mechanisms associated with a-synuclein mediated neurotoxicity [^14,15,16^] and reversed pathological and clinical progression in mice that develop basal forebrain cholinergic degeneration and behavioral deficits consistent with cholinergic dysfunction [^17^]. Further, in an exploratory 91-patient, 16-week, phase 2a clinical study in patients with DLB, neflamapimod at a dose of 40mg TID (and not at 40mg BID) improved, compared to placebo, dementia severity (assessed by Clinical Dementia Rating Sum of Boxes, CDR-SB) and gait (assessed by Timed Up and Go, TUG, test) [17,^18^], effects that were most prominent in patients with low levels of plasma ptau181 [10].

Based on those initial clinical trial results, we conducted a randomized phase 2b trial of neflamapimod in patients with DLB, selected using a ptau181 level, to enriched for participants without significant AD co-pathology to confirm phase 2a findings by assessing the effects of neflamapimod 40mg TID versus placebo over 16 weeks.

## METHODS

### Trial Design and Oversight

The RewinD-LB clinical study [ClinicalTrials.gov: NCT05869669, registered 17 May, 2023; EudraCT Number: 2023-504373-20] was a 43-site, 16-week randomized (1:1) clinical trial of neflamapimod 40mg capsules compared to matching placebo capsules, followed by a 32-week extension in which all participants received neflamapimod.

An independent Data Safety Monitoring Board approved the protocol and statistical analysis plan (SAP) and conducted safety reviews. Local and national regulatory approvals were obtained in the US, Netherlands, and UK, and all participants provided written informed consent. The study followed the Declaration of Helsinki and ICH Good Clinical Practice guidelines.

### Patients

Eligible participants were ≥55 years old and met consensus clinical criteria^19^ for probable DLB. To reduce enrollment of patients with Alzheimer’s co-pathology, individuals with elevated plasma pTau181 were excluded. The protocol originally used a ≥2.4 pg/mL cutoff based on the Simoa^®^ pTau-181 V2.0 assay. However, because the V2.0 assay became unavailable at study initiation, the V2.1 assay was used instead, and the exclusion cutoff was adjusted to ≥27.2 pg/mL, based on manufacturer guidance at the time that 27.2 pg/mL in V2.1 corresponded to 2.4 pg/mL in V2.0.

### Treatments

Once eligibility was confirmed, participants were stratified by background dementia therapy [no cholinesterase inhibitors, cholinesterase inhibitors alone, or memantine therapy (with or without concomitant cholinesterase inhibitor therapy)] and randomly assigned utilizing an Interactive Voice Recognition System to neflamapimod matching placebo capsules (1:1 ratio), TID with meals, for the 16-week randomized phase.

### Outcome Measures

The primary outcome measure was change in the CDR-SB [^20,21^]. Secondary outcome measures included the clinical Alzheimer's Disease Study Group Clinical Global Impression of Change (“CGIC”), another global measure, the Timed Up and Test (TUG) to assess motor function, and a Neuropsychological Test Battery (NTB). The NTB was a study-specific cognitive test battery that was designed to assess the following cognitive domains: attention (Identification, Detection tests), executive function (One Back accuracy), and visual learning (One Card Learning); the results of which are reported as a composite z-score. These measures were assessed at baseline, weeks 8, 12 and 16.

Plasma samples for protein biomarkers were obtained at baseline and at week 16; and on an exploratory basis levels of glial fibrillary acidic protein (GFAP) and Neurofilament light chain (NfL) were determined at the neurochemistry laboratory at Amsterdam University Medical Center using the Simoa Neurology 4-PLEX E (N4-PE) Advantage kit on the Simoa platform (Quanterix, Billerica, USA).

Structural and functional MRI were conducted at baseline and at week 16, but only at sites in the UK and the Netherlands. Images were analyzed and basal forebrain volumes and other structural and functional measures were determined utilizing a published technique^22^ at the Amsterdam Medical Center. Herein, we report the basal forebrain volume results, which had been in the protocol as the one MRI measure that would be evaluated. The remaining MRI results will be published separately.

Pharmacokinetic assessments consisted of a trough (morning pre-dose) plasma drug concentration determination at week 2. Neflamapimod drug concentration was determined by a validated liquid chromatography-tandem mass spectrometry method at Charles River Laboratories Edinburgh Ltd.

### Statistical Analyses

Per the protocol, all outcomes except the ADCS-CGIC (analyzed by two-sample tests) in the randomized phase were evaluated using a linear mixed-effects model (LME) for repeated measures which assesses the average treatment difference across all time points within each treatment period rather than only at the week 16 endpoint. Trial simulations based on phase 2a results indicated that 80 participants per arm would provide >95% power to detect a treatment effect on the primary endpoint, change in CDR-SB.

All primary analyses followed a prespecified SAP. By protocol the main clinical trial data were analyzed in December 2024 while the extension phase was ongoing. Exploratory analyses, including subset analyses by ptau181 subsets were contained in an SAP addendum that was implemented after this primary analysis, but ahead of the extension phase data being available. The primary objective of this addendum was to add in analyses of the extension phase that compared outcomes with respect to the two different batches of neflamapimod capsules used in this study, one that was the one used in both the main trial and in extension, and one that was utilized only during the extension. In the current report, results from the main randomized, double-blind phase of the trial are provided. Results of the extension phase will be reported in a separate publication, though a limited set of analyses from this phase are reported in Discussion.

For the exploratory analysis by plasma p-tau181 subset, a mixed effects model for repeated measures (MMRM) with baseline value, visit*treatment, sex and age as covariates was utilized. MMRM, rather than LME was utilized because within the randomized phase the treatment effect was not linear, with specifically the week 12 results not being consistent with week 8 and 16 results.

## RESULTS

### Patients

From August 2023 through June 2024, a total of 159 patients were randomized, with 79 patients assigned to neflamapimod and 80 to placebo (Fig 1, Consort Flow Diagram). There were no differences in baseline characteristics between those randomized to neflamapimod and those assigned to placebo (Table 1).

### Pharmacokinetics

The mean trough neflamapimod drug concentration (C_trough_; N=53 with available C_trough_ data) at steady state was 4.15 (standard error=0.21) ng/mL, which was below the expected mean 5.0 ng/mL for neflamapimod 40mg TID derived from a population PK analysis of prior clinical studies. Rather, the mean C_trough_ in the current study approached the mean of 3.7 ng/mL seen with 40mg BID in prior clinical studies.

### Primary Analysis of Clinical Endpoints

On the primary endpoint (change in CDR-SB over 16 weeks of the placebo-controlled period), no overall difference was seen between neflamapimod and placebo (Table 2). Similarly, there were no differences between placebo on the secondary clinical outcome measures (Table 2).

### Protein Biomarker and Structural MRI Results

No differences between placebo and neflamapimod were evident on change from baseline to week 16 in plasma GFAP or NfL (Figure 2A and 2B). In the MRI sub-study, there was an statistically increase in right (Fig 2D), but not left (Fig 2C) basal forebrain volume with neflamapimod treatment, while there was a decline in placebo recipients (right forebrain volume: +3.5% with neflamapimod, −4.2% with placebo, p=0.028 for the difference).

### Exploratory Post-Hoc Analyses

The original protocol specified exclusion of patients with screening plasma p-tau181 ≥2.4 pg/mL. However, because the original assay was no longer available at study initiation, and manufacturer guidance indicated that 27.2 pg/mL in the assay used in the current study corresponded to 2.4 pg/mL in the prior assay, the exclusion threshold was revised to ≥27.2 pg/mL After study completion, additional calibration data became available showing that 2.2 and 2.4 pg/mL in the original assay corresponded to approximately 21 and 23 pg/mL, respectively, in the current assay. To assess the impact of having used a screening p-tau181 threshold that was effectively higher than intended, treatment effects were evaluated in three prespecified screening plasma p-tau181 subsets: <25.2 pg/mL (equivalent to 2.6 pg/mL in the original assay), <23 pg/mL (2.4 pg/mL), and <21 pg/mL (2.2 pg/mL).

As shown in Table 3 and Figure 3A, neflamapimod demonstrated a trend toward improvement versus placebo in change from baseline in CDR-SB at Week 16 in the <23 pg/mL subset (MMRM treatment difference: −0.51; 95% CI, −1.06 to 0.13), with a larger treatment effect observed in the <21 pg/mL subset (−0.70; 95% CI, −1.42 to 0.01; p=0.054).

### Pharmacokinetic-Pharmacodynamic (PK-PD) Analyses

Identification of a potentially treatment-responsive subgroup provided an opportunity to assess the impact of the lowerthan-expected plasma neflamapimod concentrations. Specifically, in participants with screening plasma p-tau181 <21 pg/mL, change in CDR-SB was compared between neflamapimod-treated participants with trough concentrations (C_trough_) above versus below the median (3.64 ng/mL) and placebo recipients. As shown in Figure 3B, there was progressively less worsening (increase) in CDR-SB across placebo, neflamapimod participants with C_trough_ below the median (N=18 with available Ctrough data and screening p-tau181 < 21 pg/mL), and those with C_trough_ above the median (N=17), p=0.038, Jonckheere–Terpstra trend test.

### Safety

The incidence of discontinuation due to treatment-emergent adverse events (TEAEs) was low: 2.5% (2 of 79) with neflamapimod and 1.3% (1 of 80) with placebo. Of the discontinuations due to TEAEs, one was considered related, which was abnormal liver function test in a neflamapimod recipient. This participant had elevations of alanine and aspartate amino transferases to 5 times the upper limit of normal, which was not associated with bilirubin elevation and normalized with treatment discontinuation. The overall incidence of TEAEs (Table 4) was also low (only falls seen at >10% incidence overall), with no differences between treatment arms.

## DISCUSSION

The primary hypothesis that 40 mg TID of neflamapimod would improve CDR-SB outcomes versus placebo, was not confirmed in the placebo-controlled phase. However, the plasma drug concentrations obtained during that phase were lower than expected with neflamapimod dosing regimen of 40mg TID, with the mean of 4.2 ng/mL seen in that phase being close to the mean of 3.7 ng/mL seen with 40 mg BID, a dosing regimen that proved ineffective in the phase 2a clinical trial in DLB [17]. In addition, the impact of the lower-than-expected plasma drug concentrations was accentuated by the inclusion of a higher-than-expected proportion of patients with AD co-pathology, that resulted from the change in ptau181 assay and the uncertainty on the conversion between the two assays. As a result of the combination of these two issues, we believe the primary hypothesis that 40mg TID of neflamapimod would impact CDR-SB in patients who do not have substantial AD co-pathology may not have been effectively evaluated in the study.

Regarding the impact of AD co-pathology, in our previous phase 2a trial, the clinical benefit of neflamapimod was most evident in patients with a relative absence of AD co-pathology (“pure” DLB), defined by screening plasma p-tau181 <2.2 pg/mL, whereas little to no clinical activity was observed in patients with plasma p-tau181 ≥2.2 pg/mL. The 2.2 pg/mL threshold was based on published data identifying this value as the optimal (Youden) cutoff to confirm the diagnosis of AD dementia using the assay employed in that study. Published data available when the current study was designed indicated that 2.4 pg/mL was the optimal plasma p-tau181 cutoff for identifying AD co-pathology by PET imaging in patients with DLB. Accordingly, the original protocol specified exclusion of patients with plasma p-tau18 ≥2.4 pg/mL. However, because the original assay was no longer available at study initiation, and manufacturer guidance indicated that 27.2 pg/mL in the assay used in the current study corresponded to 2.4 pg/mL in the prior assay, the exclusion threshold was revised to ≥27.2 pg/mL. We had originally expected enriched recruitment into the study of participants below this p-tau181 threshold to result in at least 80% of participants not having AD co-pathology. However, based on the data from the first large (N=1298) study [^23^] that evaluated the p-tau181 assay utilized in the current study to identify AD pathology (assessed by CSF criteria), we estimate that our exclusion criteria of ≥27.2 pg/mL led to only 60–65% of our study population being free of substantive (positive CSF markers, e.g., raised p-tau181/Ab42 ratio) AD co-pathology, while use of the other cutoffs to define the patient subsets lead to progressively higher proportions of “pure” DLB: ≥25.2 pg/mL (65–75%), ≥23 pg/mL (75–80%), ≥ 21 pg/mL (80–90%). Importantly, in this larger dataset, 21 pg/mL in current version of the assay corresponds to 2.2 pg/mL in the version utilized to evaluate the phase 2a study, as well to the 2.155 pg/mL cutoff reported in the publication as the high sensitivity cutoff for AD pathology (C. Teunissen, senior author in the publication, personal communication). With this enrichment there is a progressive increase in the magnitude of the treatment effect, with the treatment effect on change in CDR-SB (−0.70 difference to placebo) in the < 21 pg/mL ptau181 subset corresponding with the phase 2a findings (difference to placebo −0.60) in the participants with screening plasma p-tau181 <2.2 pg/mL. Otherwise, the consistency of the association between the treatment effect and the stringency of the p-tau181 cutoff, combined with the biological rationale for neflamapimod being most active in the pure DLB patient population [10], supports that the hypothesis that the potential treatment effect that has been identified is in line with neflamapimod's mechanism of action with respect to the cholinergic system [14,17] and that the difference between placebo and neflamapimod is more likely to be due to a drug effect, rather than one due to chance.

The pharmacokinetic-effect analyses show that lower-than -expected plasma drug concentrations were achieved during placebo-controlled phase, and this may have contributed to the failure of the study to achieve its objectives. First, within the neflamapimod-recipients in the clinical trial who were in the upper half of trough plasma drug concentrations and had low likelihood of having AD co-pathology (based on having screening plasma p-tau181 < 21 pg/mL), mean change in CDR-SB showed slight improvement over 16 weeks, rather than the worsening seen in the placebo group, effectively matching what we had been targeting and expecting based on the phase 2a findings. A second opportunity to assess the impact of drug exposure arose during the extension phase, when a substantial subset of participants received neflamapimod capsules from a different manufacturing run (“Batch B”, versus “Batch A”, the batch of capsules utilized during the randomized phase; Fig 1). During the 1st 16 weeks of the extension phase, 55 participants received Batch A during that entire study period, and 94 participants received DP Batch B for all or a portion of those 16 weeks (46 starting on day 1, 22 starting at week 4 and 26 starting at week 8 of the Extension; Fig S1). Batch B produced plasma concentrations consistent with those expected for 40 mg TID (mean steady-state C_trough_ 5.2 [0.22]ng/mL; p=0.003 vs. concentrations during the randomized phase; Fig S2). Further, during the first 16 weeks of the extension phase, Batch B recipients showed greater improvement in CDR-SB than participants receiving the original capsules (mean difference −0.57; 95% CI, −0.98 to −0.16; Fig S3). Similarly, at Week 8, Batch B recipients had better CGIC scores (4.02 vs. 4.42; Fig S3), corresponding to a mean difference of −0.40 (95% CI, −0.77 to −0.04). Thus, the findings with Batch B during the extension demonstrated that the capsules used during the placebo-controlled phase had lower plasma drug concentrations (i.e., low bioavailability) and that higher drug exposure was associated with greater clinical activity, further supporting that the batch of capsules utilized in the main clinical trial may have underperformed based on having insufficient and aberrantly low bioavailability.

From a drug development standpoint, the reduced clinical and biological activity of the main batch of capsules, associated with a median Ctrough of approximately 4 ng/mL, i.e., only half of participants achieved Ctrough of 4 ng/mL, aligns with prior PK–PD data in early AD showing a threshold C_trough_ on an individual participant basis of 4 ng/mL below which clinical activity is limited (Fig S4) [^24^]. In contrast, 75% of participants receiving Batch B achieved the target plasma drug concentrations. These findings are also in line with *in vitro* and clinical evidence indicating that single-digit ng/mL concentrations fall within the steep segment of the sigmoidal concentration–effect curve (Fig S5). Of note, the sponsor, CervoMed Inc. has identified that the differences in plasma drug concentrations associated with the two batches can be accounted for by batch-to-batch variability in the physical form (specifically, polymorphic crystal forms) of neflamapimod [^25^], which led to differences in solubility, and therefore differences in absorption of the drug from the gut. As a result, each batch had its own intrinsic level of bioavailability that translated into consistently lower plasma drug levels with the batch of capsules utilized in the main trial, effectively allowing for an exploration of “dose-response” based on mean plasma drug exposures achieved with the two batches. Based on the results of the current trial, the sponsor will conduct future clinical trials of neflamapimod in DLB with a dose, 50mg TID, of a novel formulation that will achieve the C_trough_ target of 4 ng/mL in approximately 90% of participants [25].

Results in the placebo arm are consistent with both the clinical impression and prior published data that the clinical symptoms evolve (i.e., progress) more rapidly in DLB than in AD [^26^] and within six months [^27^]. This provided the opportunity to demonstrate potential clinical effects within a relatively short timeframe. Equally important, the potential effects seen in the < 21 pg/mL subset is the result of enriching for “pure DLB” patients, which matches the proposed mechanism of action of the drug of acting on the basal forebrain cholinergic system, with clinical disease expression in these individuals with pure DLB being predominantly driven by cholinergic functional deficits rather than neurodegenerative changes elsewhere, including entorhinal cortex-hippocampal regions which are more typical in DLB individuals with AD co-pathology.

While because of the sample size the basal forebrain results should be interpreted with caution, it should be noted that the beneficial impact of neflamapimod treatment on basal forebrain volume is also consistent with its mechanism of action and preclinical data that demonstrates a specific effect on the basal forebrain cholinergic system, including an increase in choline acetyltransferase (ChAT)-expressing neurons in the basal forebrain, an improvement in the morphology of ChAT-expressing neurons, and a restoration of function in tests of behavior associated with cholinergic function (e.g. novel object recognition) [17]. In addition, a positive treatment effect of neflamapimod on basal forebrain volume and its functional connectivity was demonstrated in a prior pilot study in patients with early AD, though that study was not placebo-controlled [18]. Further, the clinical finding of an increase in basal forebrain volume by MRI and the preclinical finding of a reversal of the loss of cholinergic neurons are consistent with a series of observations in the literature [^28,29,30^] that disease-associated basal forebrain atrophy is as much due to loss of cholinergic phenotype and individual cell shrinkage, rather than frank neuronal death and loss. Regarding the laterality of treatment effect in the current study, aside from being the result of the sample size, one potential explanation is that the severity of neurodegeneration in DLB the left basal forebrain is greater than on the right side in DLB [^31,32^], which may lead to the left side having less of a reversible component.

Our results are in line with a growing body of evidence [20,21] that support the CDR-SB as the primary outcome measure in proof-of-concept and potentially registrational studies in DLB. For example, in the NACC database, CDR-SB effectively captures clinical progression [^33^], particularly in DLB without AD co-pathology [21], where MMSE does not change over time, a difference attributed to functional impairment being captured by the CDR-SB but not by the MMSE. In line with those results, the cognitive test battery in our study showed a low level of deficit at baseline and very little change over the course of the study. Our results also show the limitations of assessing treatment effects in patients with DLB using plasma GFAP and NfL. Baseline elevations, particularly for NfL, were minimal to modest and highly variable, and the standard deviation for change over 16 weeks was several fold-higher than the mean elevation over healthy controls [23], which otherwise defines the maximal potential treatment effect.

The present study has several limitations. First, although adequately powered based on the preceding phase 2a trial, the study's ability to detect a treatment effect was reduced by the two unanticipated factors discussed earlier in this section: enrollment of a greater proportion of participants with Alzheimer's disease co-pathology than intended because of uncertainty in calibration between plasma p-tau181 assay versions, and lower-than-expected systemic exposure resulting from reduced bioavailability of the neflamapimod drug product used during the randomized phase. Second, the subgroup analyses based on plasma p-tau181 and plasma drug concentrations, though utilizing cut-offs that either developed independently in the literature (for p-tau181) or simply the median value (for C_trough_), the analyses were conducted after unblinding and analysis of the main study and should therefore be as hypothesis generating. Third, the MRI analyses were limited to a small subset of participants from sites in the United Kingdom and the Netherlands, reducing statistical power and limiting conclusions regarding treatment effects on basal forebrain structure. Finally, the randomized treatment period was limited to 16 weeks. Although this duration was selected based on prior evidence that clinical progression in DLB can be detected over this timeframe and on the positive findings from the earlier phase 2a study, longer-duration studies will be important to determine whether the observed biological and exploratory clinical effects translate into sustained clinical efficacy.

In conclusion, the phase 2a clinical study findings were not replicated, potentially due to the inclusion of a higher proportion of patients with AD co-pathology than targeted and the use of neflamapimod drug product that did not achieve expected plasma drug concentrations. The full study data combined with the phase 2a results indicate that further clinical evaluation of neflamapimod in a more stringently defined population of DLB without AD co-pathology and with neflamapimod drug product that achieves targeted plasma drug concentrations is warranted.

## Supplementary Material

Supplementary Files

This is a list of supplementary files associated with this preprint. Click to download.

- SupplementalFigures.docx

## Figures and Tables

**Figure 1:**
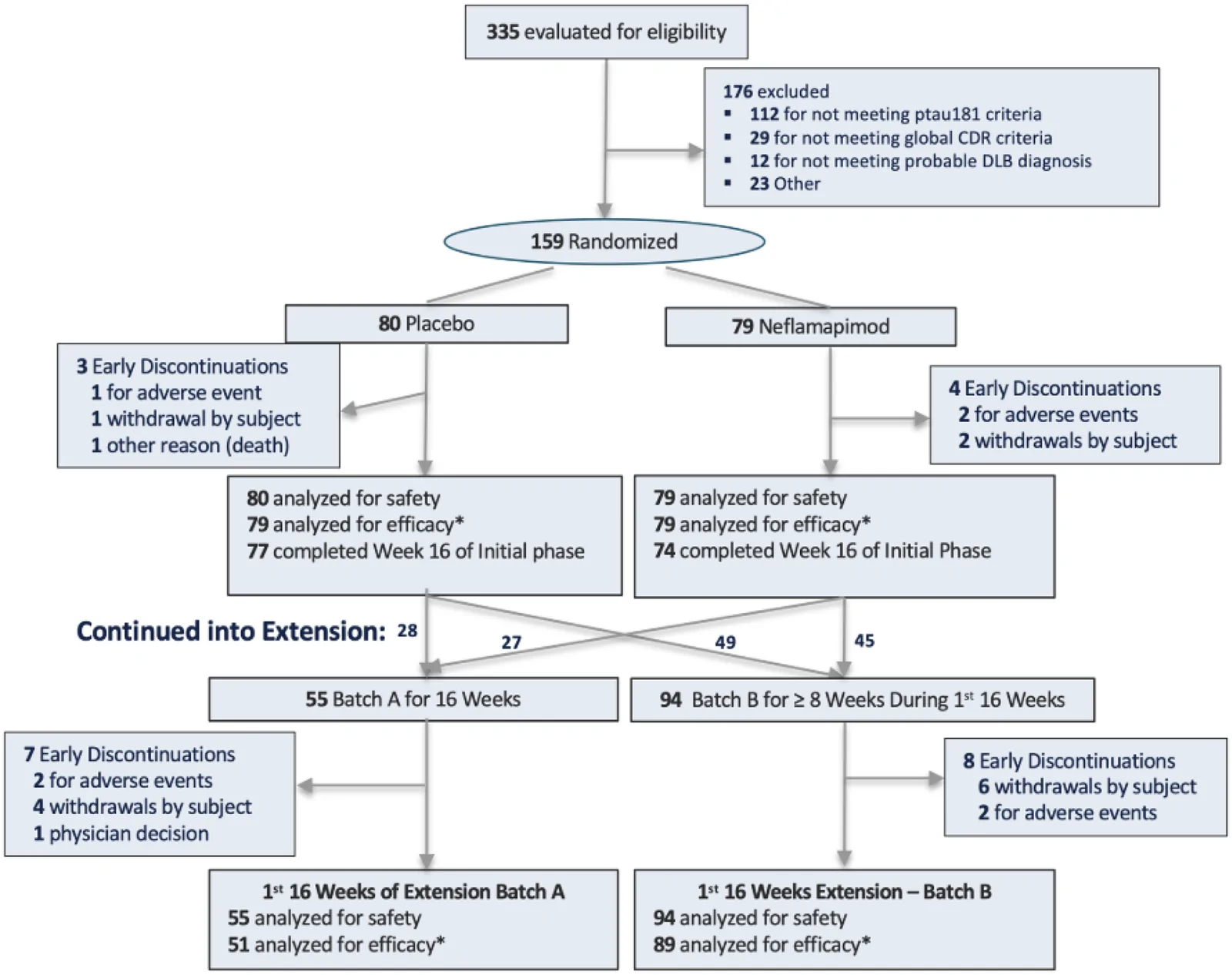
Consort Flow Diagram. Consort participant flow diagram. Batch A refers to the neflamapimod capsules utilized during the placebo-controlled period, as well by the indicated participants during the extension. Capsules from Batch B were exclusively utilized during the extension phase by the indicated participants. * - modified intention-to-treat (mITT): at least one efficacy evaluation in respective study phase required to be included in efficacy population.

**Figure 2:**
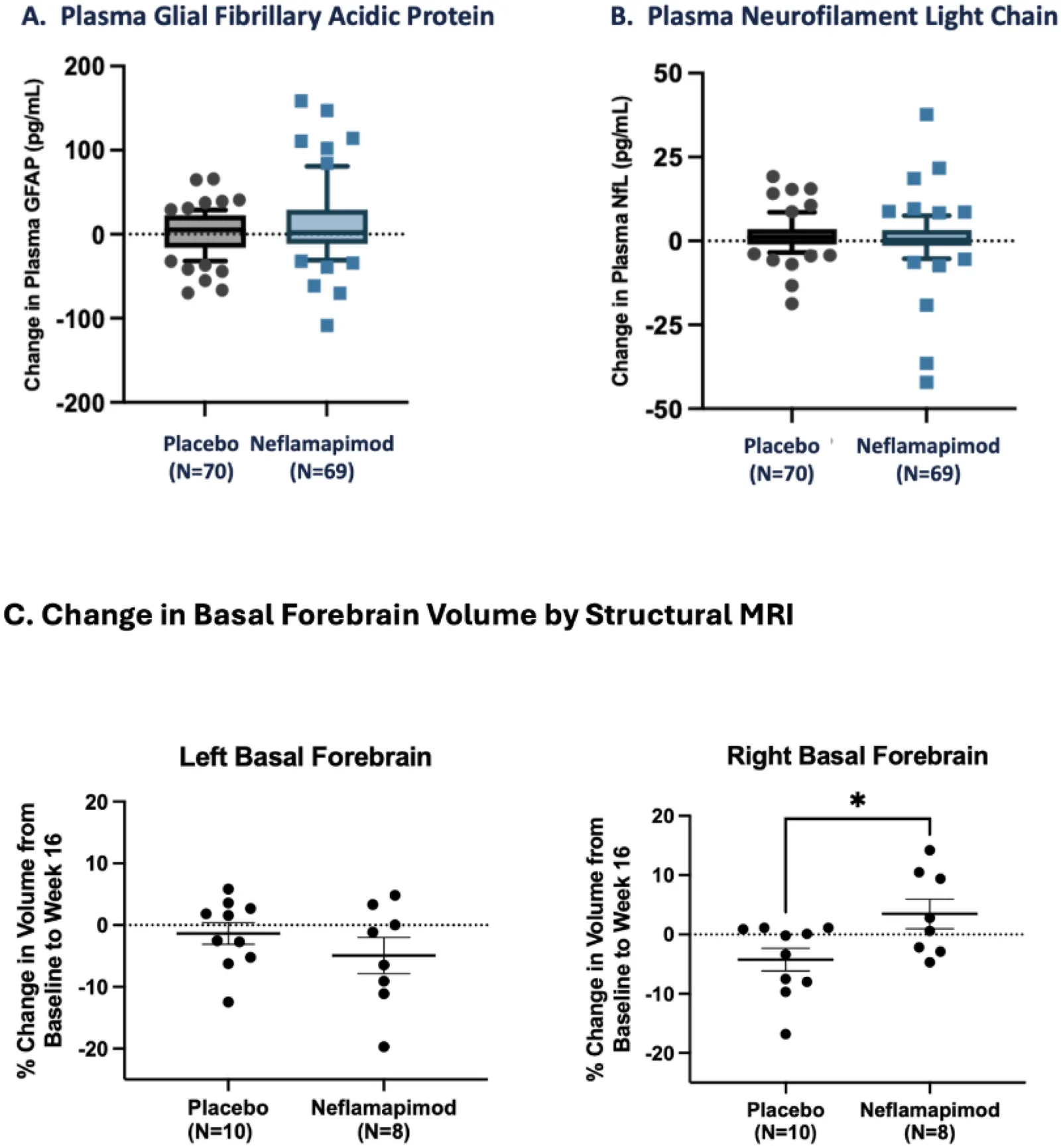
Biomarker Results A. Change (pg/mL) from baseline to Week 16 in Levels of Plasma Glial Fibrillary Acidic Protein (GFAP). B. Change (pg/mL) from baseline to Week 16 in Levels of Neurofilament Light Chain (NfL). C. Percent change from baseline to week 16 in volume by structural magnetic resonance imaging (MRI) in left (left panel) and right (right panel) basal forebrain. * *P*<0.05.

**Figure 3:**
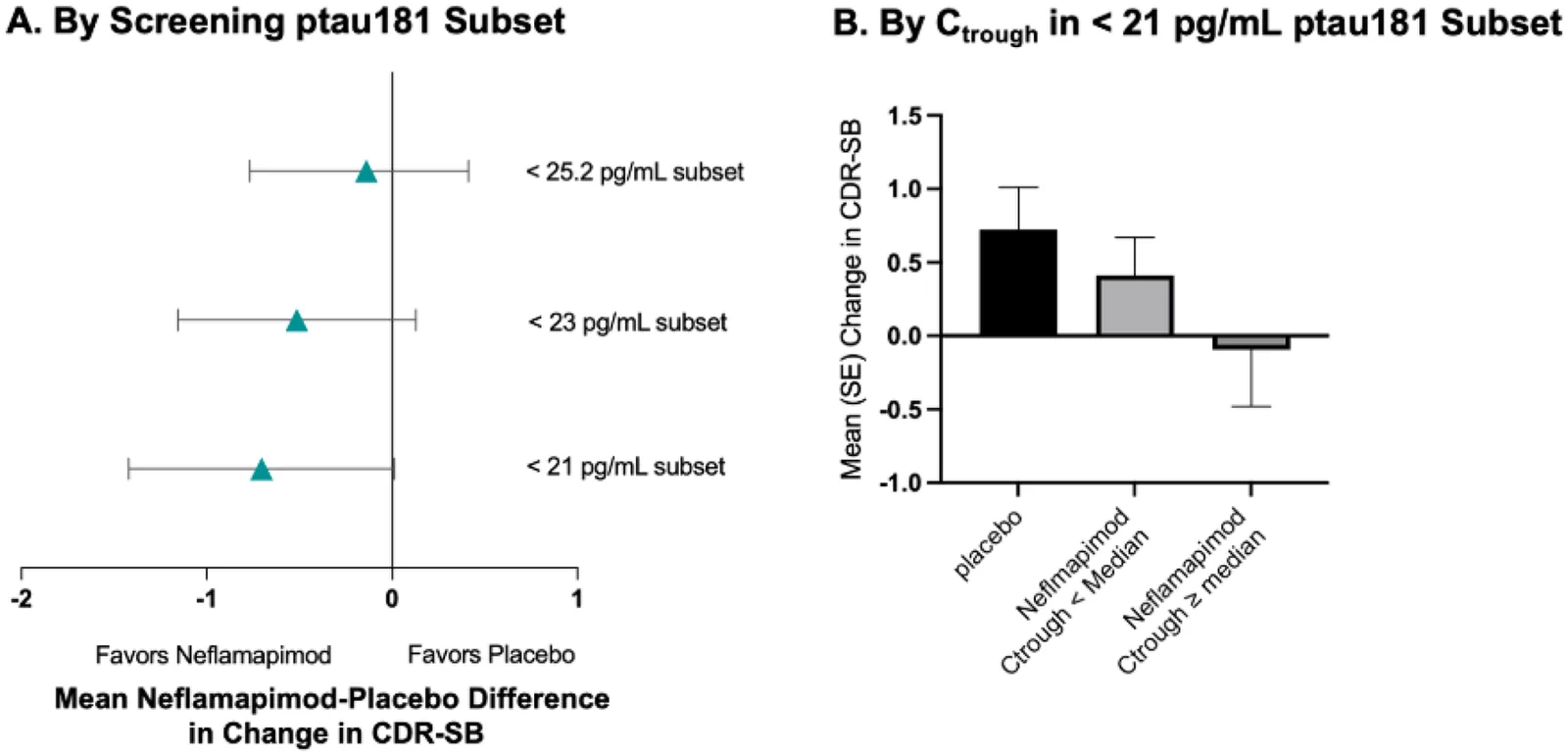
Exploratory Analyses of Change in CDR-SB over 16 Weeks by Screening Plasma pTau181 Level and Plasma Trough Drug Concentration (C_trough_) (A) Mean (95% confidence interval) difference between neflamapimod and placebo in change in CDR-SB from baseline to week 16, by plasma ptau181 subset, from mixed model for repeated measures (MMRM) analysis with baseline value, visit*treatment, sex and age as covariates. Number of subjects in each subset by treatment group are provided in Table 3. (B) Mean (standard error) change in CDR-SB from baseline to week 16 by trough plasma drug concentration (C_trough_ < or ≥ median for the study) in < 21 pg/mL p-tau181 subset. P=0.038 for progressively less worsening (increase) in CDR-SB across placebo (N=45), neflamapimod participants with C_trough_ below the median (N=18), and those with C_trough_ above the median (N=17), Jonckheere–Terpstra trend test. The number of participants in the neflamapimod group was limited to those for whom a C_trough_ measurement was available.

**Table 1.** Baseline Characteristics

	Placebo	Neflamapimod
Number of Participants	79	80
Age (Mean, SD)	70.7 (5.8)	72.1 (6.4)
Male	67 (83.8%)	69 (87.3%)
MMSE (Mean, SD)	23.3 (4.6)	23.6 (4.3)
CDR-SB (Mean, SD)	4.23 (1.71)	4.45 (2.17)
ISLT Immediate (Mean, SD)	14.8 (5.9)	13.0 (5.0)
Core Clinical Criteria:		
Cognitive Fluctuations	59 (73.8%)	59 (74.7%)
Visual Hallucinations	45 (56.3%)	43 (54.4%)
REM sleep behavioral disorder	64 (80.0%)	60 (75.9%)
Parkinsonism	68 (85.0%)	70 (88.6%)
Background Therapy:		
AChEI* alone	52 (65.0%)	50 (63.3%)
Memantine (with or without AChEI)	11 (13.8%)	12 (15.2%)
No background Therapy	17 (21.3%)	17 (21.5%)
Screening plasma ptau181 level (pg/mL; Mean, SD)	19.1 (5.2)	18.9 (4.9)
Number (%) with screening plasma p-tau181<21 pg/mL	46 (61%)	48 (60%)
Plasma GFAP (pg/mL; Mean, SD)	140.3 (71.0)	149.8 (88.4)
Plasma NfL (pg/mL; Mean, SD)	19.8 (13.1)	23.7 (13.6)
Left Basal Forebrain Volume (mm^3^; Mean, SD)	312.6 (38.9)	310.6 (33.9)
Right Basal Forebrain Volume (mm^3^; Mean SD)	324.0 (42.7)	299.0 (32.1)

**Abbreviations:** AChEI – Acetylcholinesterase inhibitors; CDR-SB – Clinical Dementia Rating Sum of Boxes; GFAP – glial fibrillary acidic protein; ISLT – International Shopping List Test; MMSE – Mini mental status examination; NfL – neurofilament light chain; ptau181 – tau phosphorylated at position 181; REM – rapid eye movement

**Table 2.** Primary and Secondary Clinical Outcome Measures

	Placebo (N=79)	Neflamapimod (N=79)
Change in Clinical Dementia Rating Sum of Boxes (CDR-SB)		
Mean (SE) Change at Week 8	0.47 (0.16)	0.39 (0.18)
Mean (SE) Change at Week 16	0.59 (0.20)	0.49 (0.20)
Mean Difference (95% CI)	−0.02 (−0.41, 0.37)	
p-value	P=0.9	
Change in Timed Up and Go (TUG)		
Mean (SE) Change at Week 8	2.37 (3.15)	−0.02 (0.45)
Mean (SE) Change at Week 16	−0.61 (0.46)	−0.07 (0.52)
Mean Difference (95% CI)	0.29 (−1.9, 2.4)	
ADCS-CGIC		
Mean (SE) Score at Endpoint	4.46 (0.12)	4.42 (0.11)
Mean Difference (95% CI)	−0.04 (−0.36, 0.28)	
Neuropsychological Test Battery (NTB)		
Mean (SE) Change at Week 8	0.05 (0.06)	0.06 (0.06)
Mean (SE) Change at Week 16	0.13 (0.06)	−0.07 (0.06)
Mean Difference (95% CI)	−0.02 (−0.12, 0.08)	

**Table 3.** Mixed-Model for Repeated Measures (MMRM) Analysis of Change from Baseline to Week 16 by Screening Plasma p-Tau181 Subset

	Least Square Mean (SE) Change at Week 16 of Randomized Phase		Treatment Effect	
Population	Placebo	Neflamapimod	Placebo-Neflamapimod Difference	95% CI
All (mITT)	0.410 (0.239) N=77	0.273 (0.247) N=74	−0.137	(−0.699, 0.425,)
p-tau181 <25.2 pg/mL	0.362 (0.282) N=68	0.223 (0.247) N=62	−0.139	(−0.769, 0.491)
p-tau181 <23 pg/mL	0.360 (0.288) N=56	−0.154 (0.306) N=54	−0.514	(−1.156, 0.128)
p-tau181 <21 pg/mL	0.660 (0.340) N=45	−0.044 (0.356) N=46	−0.704	(−1.424, 0.012)

**Table 4.** Most Common Treatment Emergent Adverse Events (TEAE)

Preferred Term	Neflamapimod (40 mg TID) (N=79) n (%)	Placebo (N=80) n (%)
Any TEAE	58 (73.4)	59 (73.8)
Fall	12 (15.2)	15 (18.8)
COVID-19	8 (10.1)	3 (3.8)
Diarrhea	6 (7.6)	3 (3.8)
Urinary tract infection	5 (6.3)	6 (7.5)
Upper respiratory tract infection	5 (6.3)	4 (5.0)
Headache	5 (6.3)	10 (12.5)
Fatigue	5 (6.3)	6 (7.5)
Dizziness	4 (5.1)	3 (3.8)
Confusional state	4 (5.1)	1 (1.3)
Alanine aminotransferase increased	4 (5.1)	1 (1.3)
Back pain	4 (5.1)	1 (1.3)
Hallucination	2 (2.5)	5 (6.3)

## Data Availability

Deidentified individual participant data from that underlies the clinical trial results reported in this article will be submitted to the clinical trial data sharing platform Vivli (vivli.org) by 12 months from publication.

## Abbreviations

AD: Alzheimer's Disease
ADCS-CGIC: Alzheimer's Disease Cooperative Study - Clinical Global Impression of Change
BID: twice a day
CDR-SB: Clinical Dementia Rating Sum of Boxes
ChAT: choline acetyltransferase
C_trough_: trough plasma drug concentration
DLB: Dementia with Lewy bodies
GFAP: Glial fibrillary acidic protein
ISLT: International Shopping List Test
LME: Linear mixed effects model
MMRM: Mixed model for repeated measures
MMSE: Mini Mental Status Examination
MRI: Magnetic resonance imaging
NACC: National Alzheimer's Coordinating Center
NfL: Neurofilament light chain
NTB: Neuropsychological Test Battery
PET: positron emission tomography
PK-PD: pharmacokinetic-pharmacodynamics
p-Tau181: tau phosphorylated at position 181
SAP: Statistical analysis plan
TID: three times a day
TUG: Timed Up and Go Test
